# Bringing the Cath Lab to the Neonatal Intensive Care Unit (NICU): A Three-Case Series About Bedside Echocardiography-Guided Transcatheter Patent Ductus Arteriosus Closure in Extreme Low Birth Weight Infants

**DOI:** 10.7759/cureus.108468

**Published:** 2026-05-08

**Authors:** Ling Ai Soon, Geraldine Chow, Su Wei Ng, Mohammad Tamim Jamil, Koh Ghee Tiong

**Affiliations:** 1 Pediatric Cardiology, Hospital Pulau Pinang, Georgetown, MYS; 2 Pediatric Medicine, Hospital Pulau Pinang, Georgetown, MYS; 3 Pediatric Cardiology, Hospital Sultan Idris Shah Serdang, Selangor, MYS

**Keywords:** bedside intervention, echocardiography guidance, interventional cardiologist, low-birth-weight neonate, neonatal intensive care unit, neonatologist, patent ductus arteriosus, pda device occlusion, preterm infant, transcatheter closure

## Abstract

Patent ductus arteriosus (PDA) remains a significant driver of morbidity in extreme low birth weight (ELBW) infants. Pharmacological closure often fails or is contraindicated, while surgical ligation carries significant perioperative and postoperative risks. Recent advances in device technology have enabled transcatheter PDA closure in very small infants, including procedures performed at the bedside in the neonatal intensive care unit (NICU).

We report a case series of three ELBW infants (gestational age 24-27 weeks; weight at procedure 1.0-1.28 kg) who successfully underwent bedside transcatheter PDA closure using the Amplatzer Piccolo Occluder (Abbott Structural Heart, Plymouth, MN) under echocardiographic guidance in the NICU. These infants remained ventilator dependent due to hemodynamically significant PDA (hsPDA) and had failed medical therapy.

Technical success was achieved in all cases without immediate device-related complications; two infants demonstrated significant respiratory improvement with successful extubation following the procedure. One infant, despite successful PDA device occlusion, passed away due to complications related to extreme prematurity, necrotizing enterocolitis, and nosocomial sepsis.

This case series highlights the feasibility, safety, and potential respiratory benefits of bedside transcatheter PDA device occlusion in ELBW infants when performed by an experienced multidisciplinary team.

## Introduction

Patent ductus arteriosus (PDA) is a common clinical condition in preterm infants [1] and is associated with multiple adverse outcomes, including bronchopulmonary dysplasia (BPD), necrotizing enterocolitis (NEC), renal impairment, intraventricular hemorrhage (IVH), and prolonged ventilator dependence. Management of PDA in preterm infants remains controversial due to the limited efficacy and potential complications of available treatment modalities. Nevertheless, it is generally agreed that closing the arterial ducts in selected infants reduces the length of ventilation and improves their neonatal course [2].

Medical management with non-steroidal anti-inflammatory drugs (NSAIDs) remains the first line of therapy with a moderate success rate in closing the PDA [3]. However, its application is often limited by clinical contraindications common in preterm populations, such as renal dysfunction, gastrointestinal complications, thrombocytopenia, or sepsis. Surgical ligation, although effective, is associated with significant perioperative and postoperative risks, particularly in extreme low birth weight (ELBW) infants. Inherent risks during transportation include cardiorespiratory instability, thermo-dysregulation, accidental endotracheal tube (ETT) dislodgement, displacement of invasive lines, infusion pump dysfunction leading to discontinuation or purge of inotropes, and incubator malfunction [4]. Besides, vocal cord paralysis, chylothorax, and potential adverse neurodevelopmental outcomes are also described in the literature.

Recent advancements in transcatheter technology, particularly the development of the Amplatzer Piccolo Occluder (Abbott Structural Heart, Plymouth, MN), have enabled PDA closure in infants weighing as little as 700 g. By moving the procedure to the bedside and utilizing real-time echocardiographic guidance, we have reduced the logistical hurdles of the catheterization lab. Ultimately, this strategy may protect the infant from the physiological demands of transport. This strategy is especially relevant for ELBW infants, a population characterized by profound cardiopulmonary immaturity, extreme vascular fragility, and limited physiologic reserve.

We report a case series of bedside transcatheter PDA closures performed on three ventilator-dependent ELBW infants at Hospital Pulau Pinang, Malaysia. Although transcatheter closure is a recognized alternative to surgery globally, bedside NICU procedures remain rare in the Malaysian clinical literature. In this case series, we highlight the feasibility and safety of bedside echocardiography-guided PDA device occlusion in carefully selected ELBW infants. 

## Case presentation

Methods

**Figure 1:**
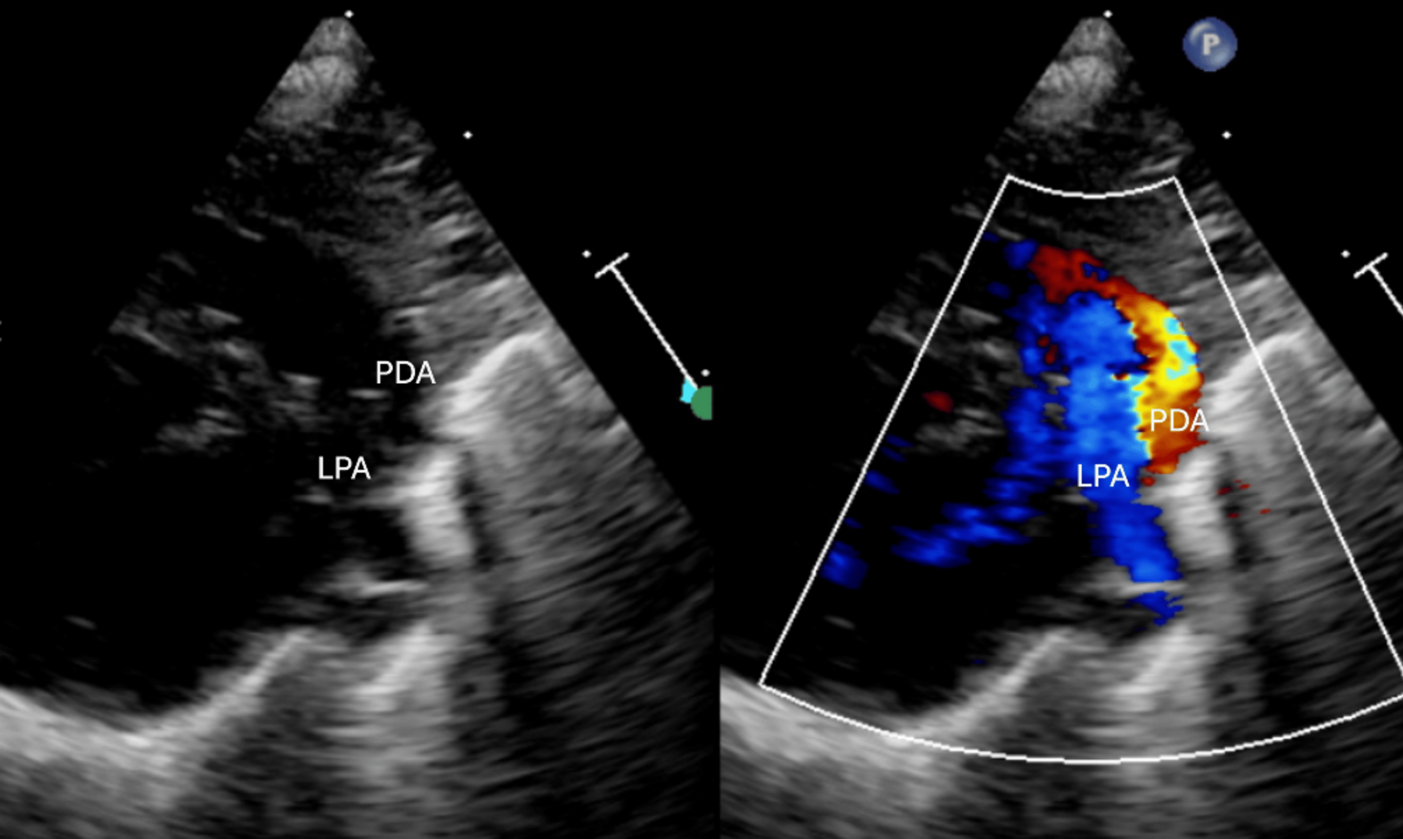
Echocardiography showed Type F PDA which characterized by a long, tortuous, and tubular morphology. Notably, it exhibits a distinct caudal angulation at the pulmonary artery insertion point.

Our case selections were based on the clinical, radiographic, and echocardiographic assessments. Eligible cases included those with fetal-type (Type F) PDA (Figure 1), left-to-right shunting, and either a left atrium-to-aortic ratio (LA/Ao) > 1.5 or clinical evidence of pulmonary overcirculation, gut, or renal hypoperfusion. First-line pharmacological therapy consisted of paracetamol at a dosage of 15 mg/kg for five days. If treatment failed after two courses, surgical ligation or transcatheter PDA device occlusion was considered.

**Figure 2:**
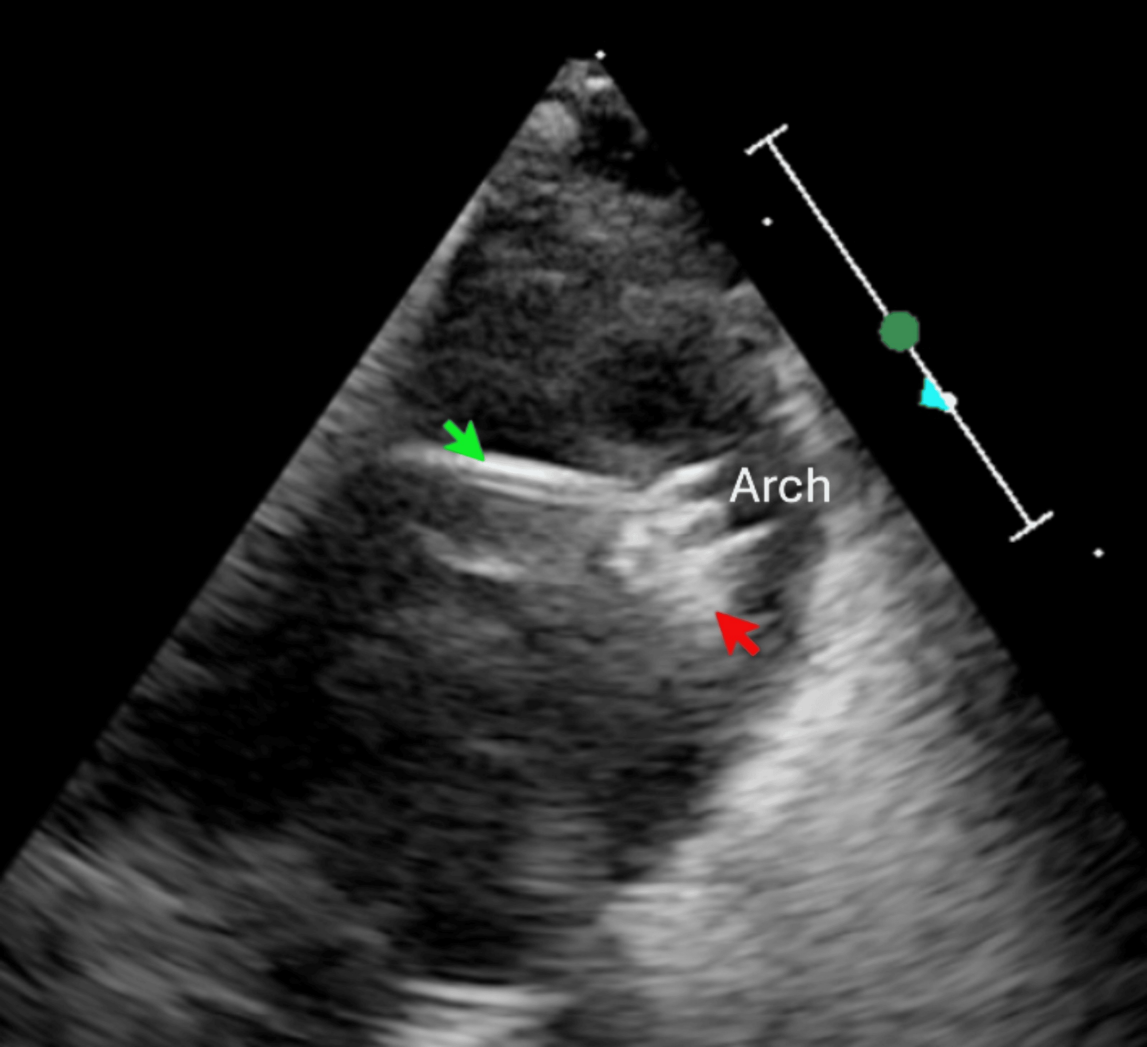
Echocardiography demonstrates the device (indicated by the red arrow) loaded within the delivery sheath (green arrow) and positioned at the patent ductus arteriosus (PDA).

**Figure 3:**
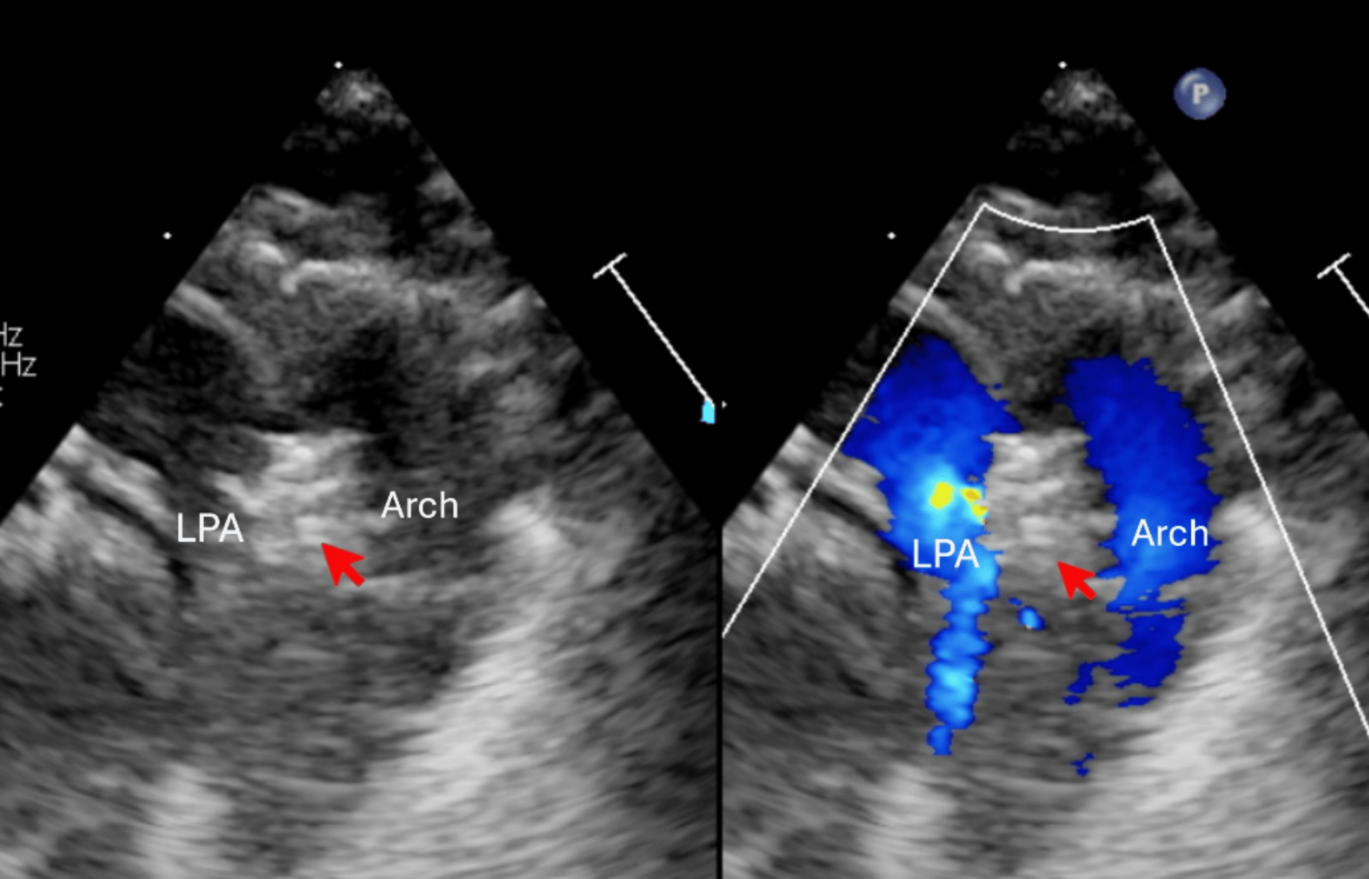
Echocardiography showed device position (indicated by red arrow) with color doppler no gradient in LPA and across the arch.

Written informed consent for the procedure was obtained from the parents of all patients. Procedures were performed under intravenous sedation, analgesia, and muscle relaxant in the NICU setting; all infants were mechanically ventilated and maintained under a radiant warmer with continuous vital signs monitoring. Under sterile conditions, ultrasound-guided access was established via the femoral vein using a 4 Fr sheath. Heparin (50 U/kg) and a single dose of cefuroxime (50 mg/kg) were administered before the procedure. Echocardiography was used throughout the procedure, including during wire insertion, device positioning (Figure 2), and deployment. Post-deployment, echocardiography was used to check for left pulmonary artery (LPA) obstruction or aortic arch obstruction (Figure 3). 

Case one

A male infant was born at 24 weeks’ gestation with a birth weight of 740 g. The mother was a known Group B Streptococcus carrier. The infant developed respiratory distress syndrome requiring a single dose of surfactant and prolonged respiratory support, including invasive mechanical ventilation for 33 days, followed by non-invasive ventilation (NIV). His clinical course was complicated by persistent ventilator dependence attributed to a hemodynamically significant patent ductus arteriosus (hsPDA).

**Figure 4:**
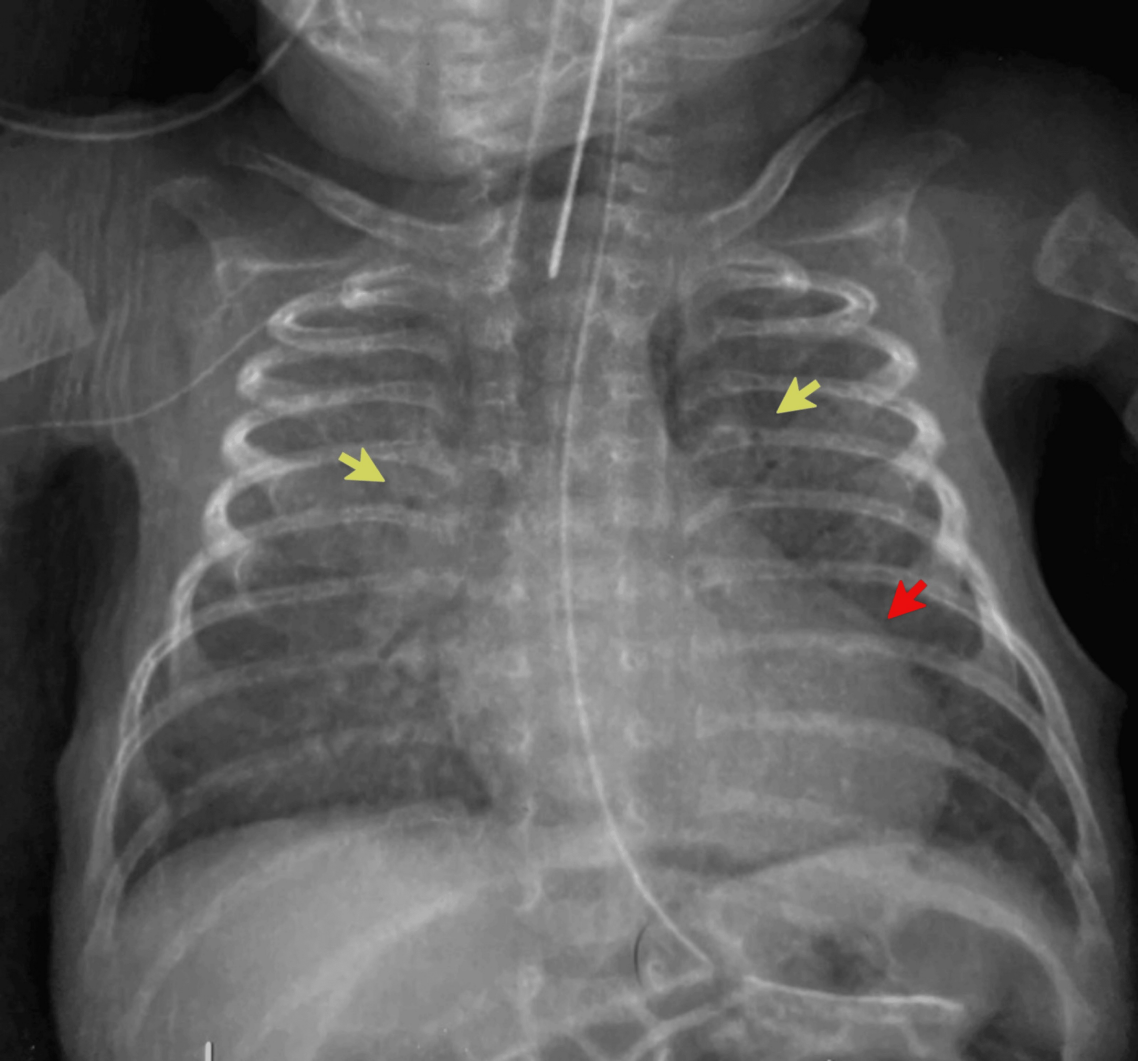
The pre-procedural anteroposterior radiograph demonstrates marked cardiomegaly (red arrow) with diffuse pulmonary overcirculation and perihilar haze (yellow arrow), suggestive of pulmonary venous congestion.

Two courses of paracetamol were administered, but failed to achieve ductal closure. Transthoracic echocardiography performed on day 33 of life demonstrated a large PDA measuring 3.0 mm at the narrowest diameter, with an ampulla of 3.0 mm and a ductal length of 7.5 mm. The left atrium and left ventricle (LA/LV) were dilated with an LA/Ao ratio of 1.6. The chest x-ray pre-procedure (Figure 4) revealed cardiomegaly with a congested lung field.

**Figure 5:**
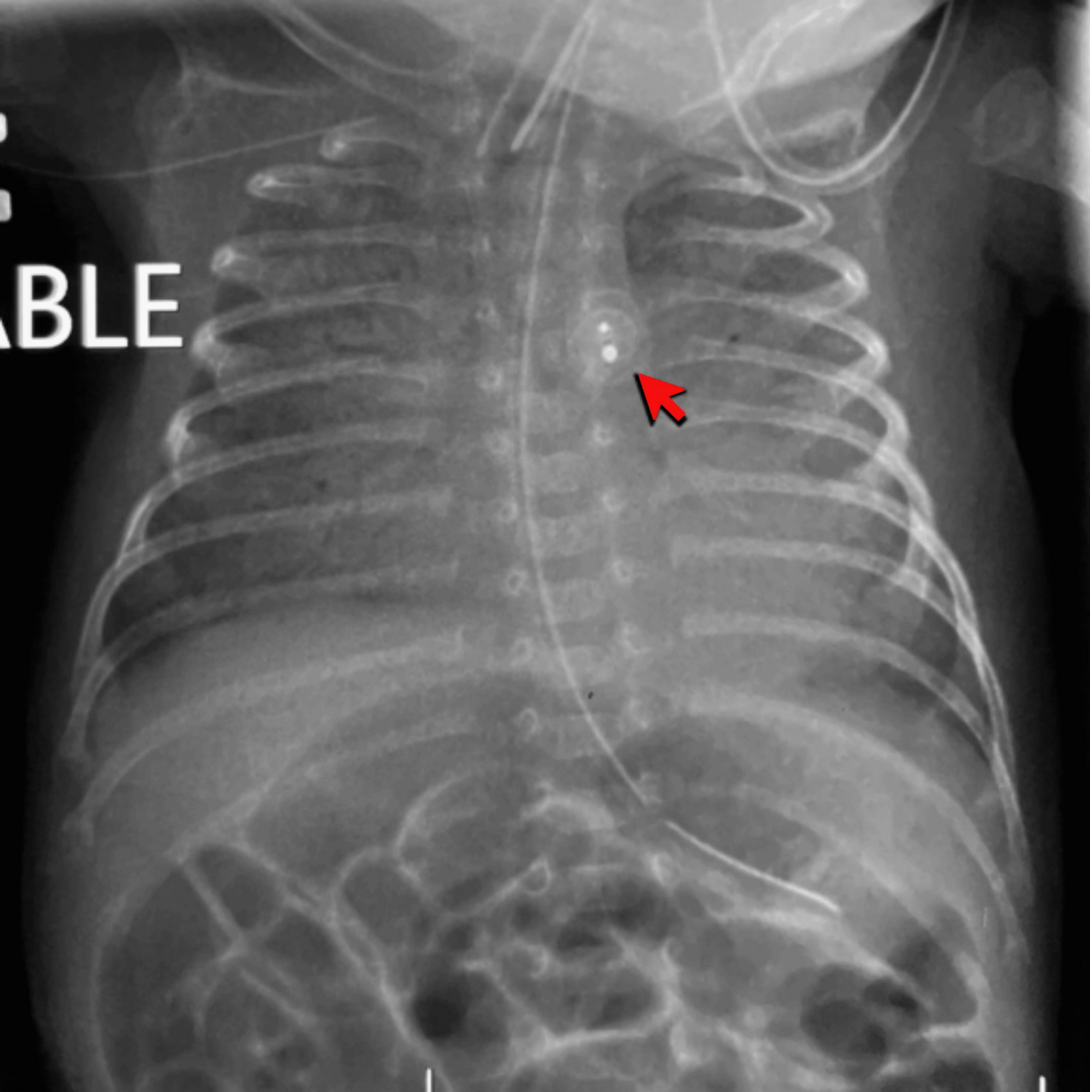
Following the procedure, the chest x-ray reveals a well-seated PDA device (indicated by the red arrow).

Given the failure of medical therapy and persistent NIV dependence, bedside transcatheter PDA device occlusion was performed on day 36 of life in NICU after discussion with parents. At the time of intervention, the infant weighed 1.0 kg. The procedure was conducted under echocardiographic guidance, and the device used was a 5/2 Piccolo Occluder device. Device deployment was uncomplicated, with immediate echocardiographic confirmation of complete ductal closure and no obstruction to the left pulmonary artery or descending aorta. The post-procedure chest x-ray (Figure 5) confirmed the position of the device.

Following the procedure, the infant demonstrated gradual respiratory improvement and was successfully extubated eight days later. No procedure-related complications were observed during follow-up, and the infant was then discharged home one month post-procedure.

Case two

A male infant was born at 24 weeks’ gestation with a birth weight of 835 g following an in vitro fertilization pregnancy. He developed severe respiratory distress syndrome requiring surfactant therapy and invasive mechanical ventilation since birth. His recovery was hindered by a hemodynamically significant PDA, leading to prolonged ventilator dependence.

**Figure 6:**
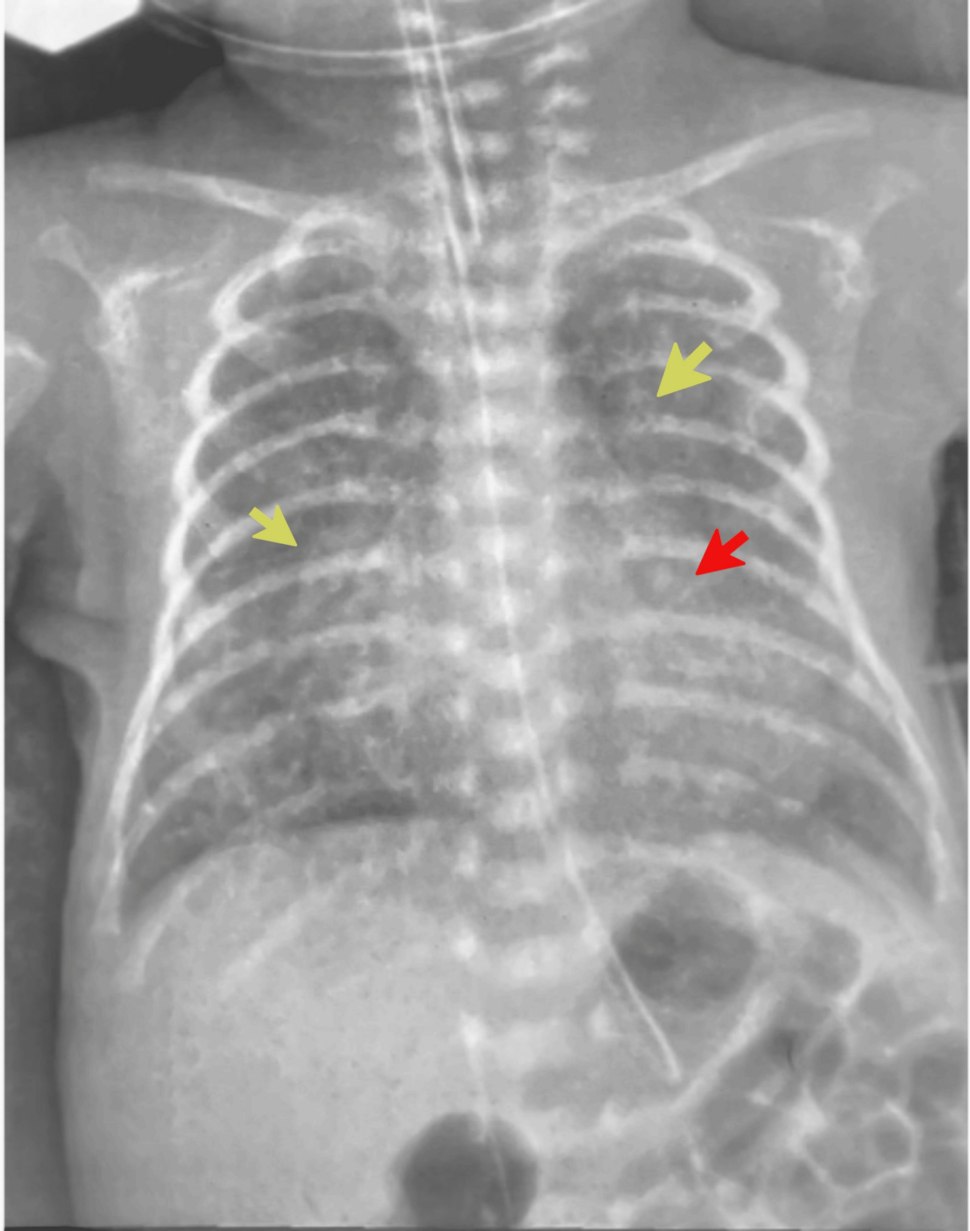
Pre-procedural chest radiography demonstrated marked cardiomegaly (red arrow) and profound pulmonary overcirculation, with bilateral diffuse opacities (yellow arrow) suggestive of pulmonary overcirculation.

Pharmacological closure with paracetamol was attempted but discontinued due to concern about poor renal clearance in this ELBW infant. Serum creatinine rose to 128 µmol/L after the second course of intravenous paracetamol, likely reflecting limited renal function, further worsened by concurrent sepsis. Ibuprofen was contraindicated. Repeat echocardiography demonstrated a large PDA measuring 2.3 mm at the narrowest diameter, with an ampulla of 3.7 mm and a ductal length of 9.6 mm. The LA/LV was dilated with an LA/Ao ratio of 1.8. The pre-procedure chest x-ray (Figure 6) revealed cardiomegaly with bilateral increased pulmonary vascular markings, suggestive of volume overload.

**Figure 7:**
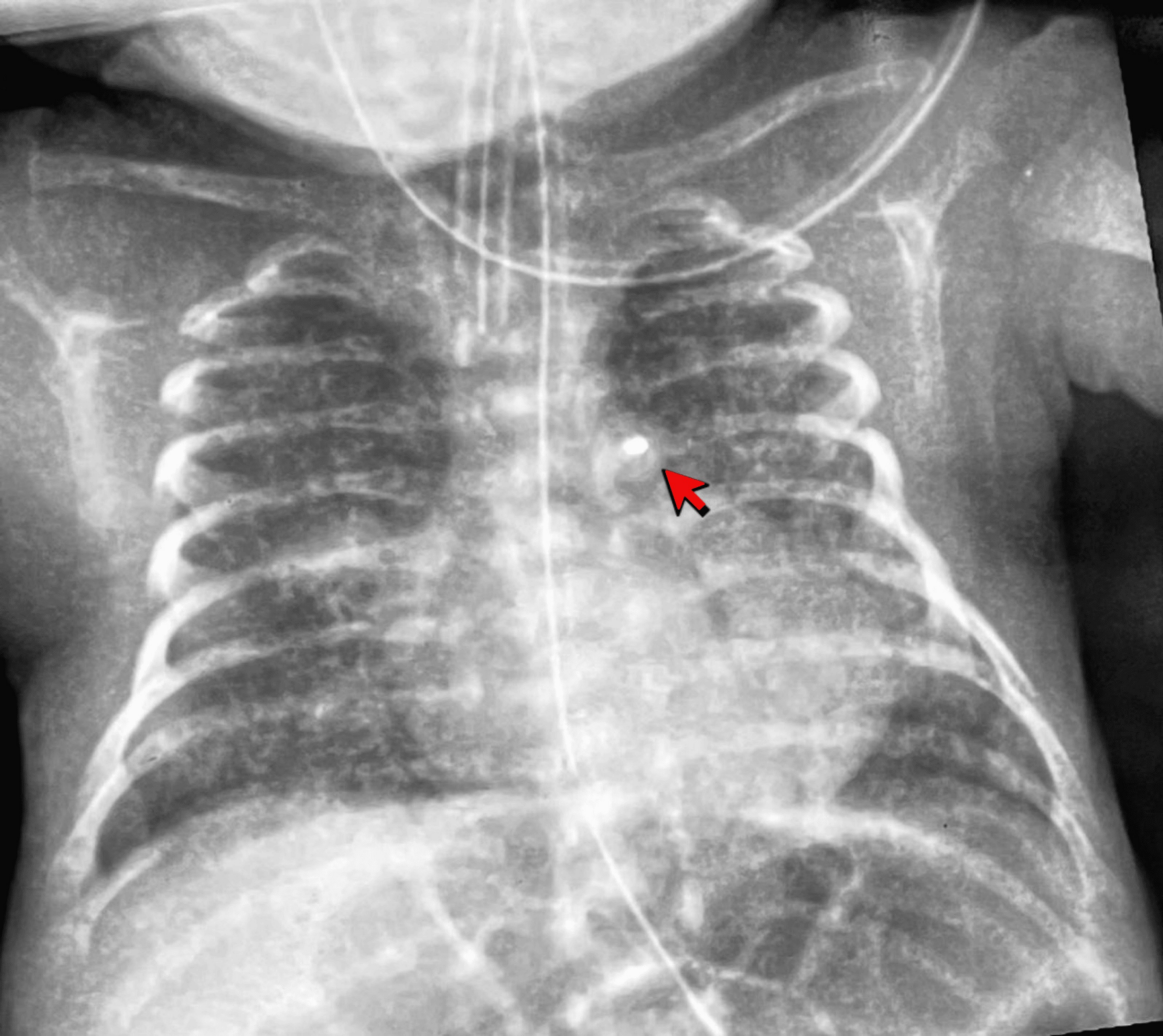
Post-procedural chest radiograph demonstrates the device in situ (red arrow). Note the interval reduction in cardiothoracic ratio and reduced pulmonary vascular marking compared to the pre-procedure chest x-ray.

Given failure of medical therapy and ongoing ventilator dependence, bedside transcatheter PDA closure was performed on day 31 of life in the NICU after thorough parental counselling. Body weight was 1.05 kg during the procedure. A 4/2 Piccolo Occluder device was selected and successfully deployed under real-time echocardiographic guidance. Post-procedural imaging confirmed complete ductal closure with no obstruction to the left pulmonary artery or descending aorta. The post-procedure chest x-ray (Figure 7) confirmed the PDA device position.

The infant demonstrated gradual respiratory improvement and was successfully extubated 15 days following PDA device closure. No immediate or delayed device-related complications were observed.

Case three

A male infant was born at 27 weeks’ gestation with a birth weight of 1.0 kg following an uncomplicated antenatal course. He required invasive mechanical ventilation immediately after birth and was escalated to high-frequency oscillatory ventilation (HFOV) on day nine of life due to worsening respiratory failure.

Echocardiography revealed a hemodynamically significant PDA (hsPDA). Two courses of paracetamol were administered, but failed to achieve ductal closure. On day 9 of life, the infant developed necrotizing enterocolitis (NEC) and was managed conservatively with bowel rest. Blood cultures remained negative throughout this period.

**Figure 8:**
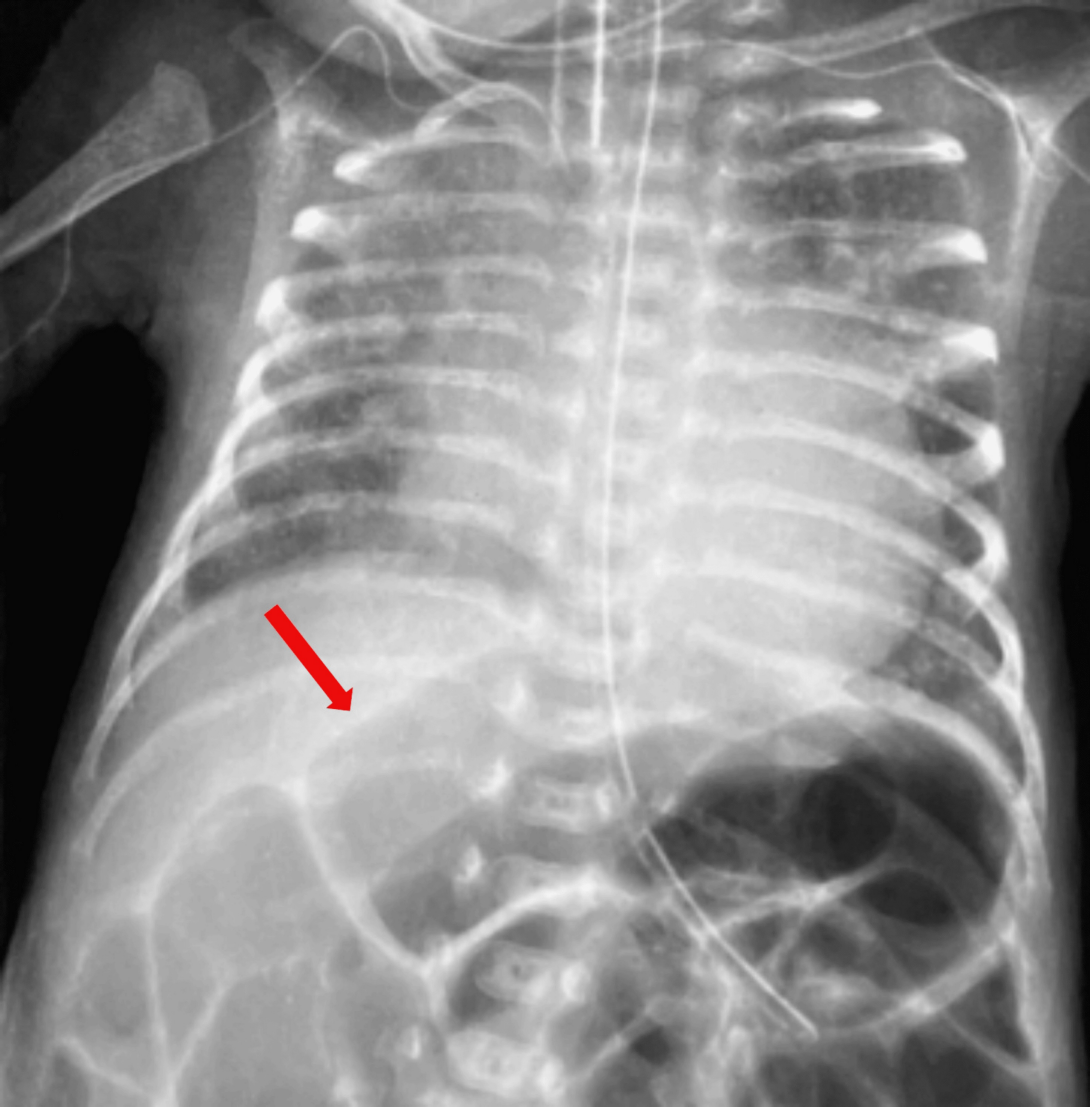
Initial chest radiography demonstrated marked cardiomegaly with a significant pulmonary plethora. Note the prominent congestive pattern across the lung fields and the presence of dilated, gas-filled bowel loops (red arrow).

Serial echocardiographic assessments demonstrated a large PDA measuring 3.0 mm in diameter, ampulla of 3.4 mm, ductal length of 6.0 mm, with a hypertensive Doppler pattern. There was dilated LA/LV with a significant LA/Ao ratio of 1.7. In view of failed medical therapy and persistent hsPDA, bedside transcatheter PDA device occlusion was proposed. Following a detailed discussion of the risks and benefits with the parents, the decision was made to proceed with the intervention. The pre-procedural chest x-ray (Figure 8) demonstrated cardiomegaly and pulmonary congestion, correlating with echocardiographic findings of a significant left-to-right shunt.

**Figure 9:**
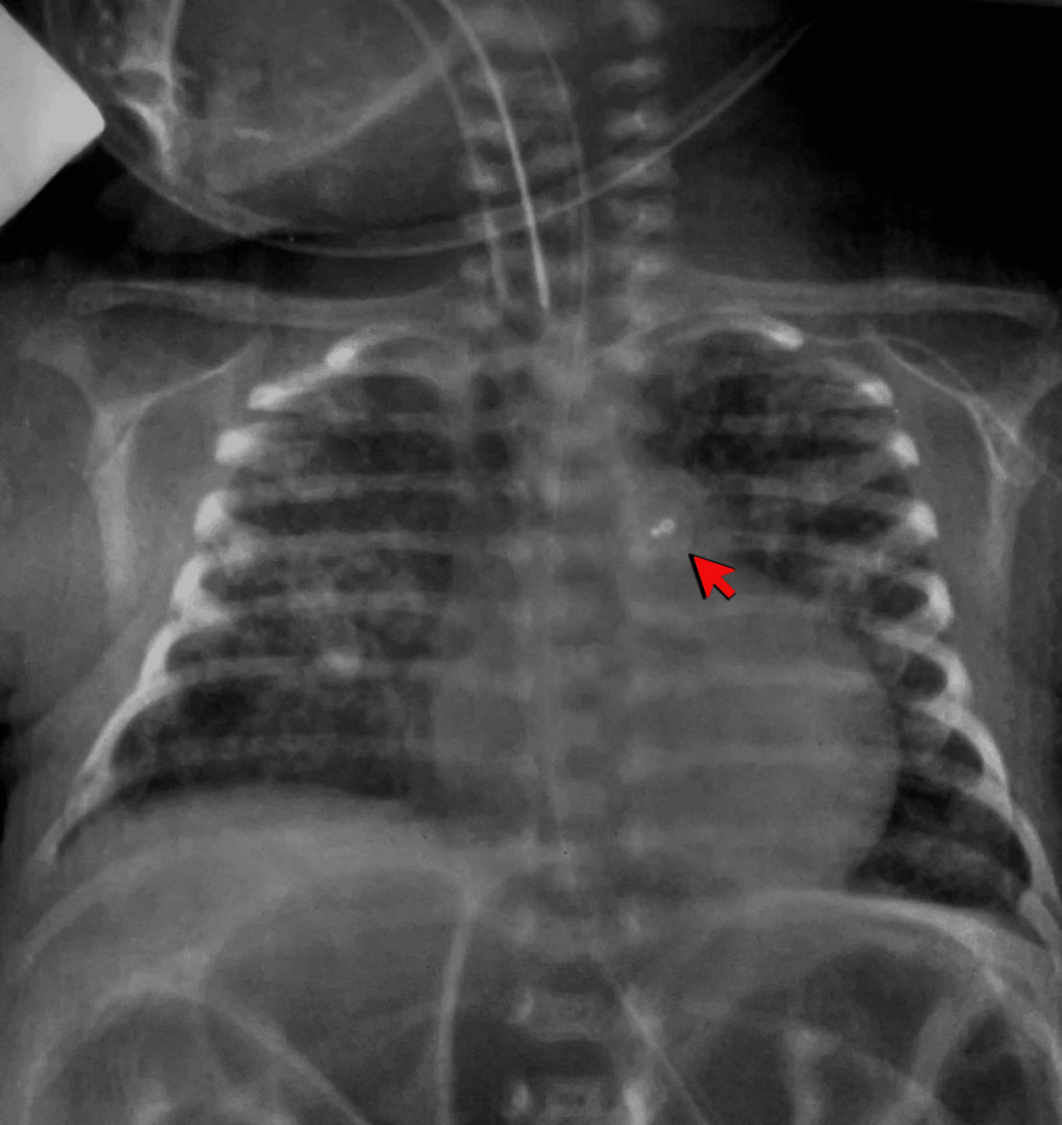
Following the procedure, the well-positioned device is clearly visible (red arrow). The lung fields appear less voluminous. The pulmonary vasculature is less congested, reflecting successful hemodynamic stabilization.

The procedure was performed on day 32 of life in the NICU using a 5/2 Piccolo Occluder device, at a body weight of 1.28 kg. Immediate post-procedural echocardiography confirmed appropriate device positioning, complete ductal closure, and absence of obstruction to adjacent structures. The post-procedure chest x-ray (Figure 9) confirmed the device position.

Despite successful PDA closure, the infant remained dependent on HFOV. Five days post-procedure, he developed progressive abdominal distension, necessitating urgent surgical evaluation. An exploratory laparotomy and contrast study revealed a hepatic flexure stricture with extensive intra-abdominal adhesions. A right hemicolectomy with ileocolic anastomosis was performed.

Although initial postoperative recovery was achieved, he later developed nosocomial sepsis. Despite maximal supportive care, his condition deteriorated, and he died on day 96 of life.

The clinical characteristics and outcomes of all three cases are summarized in Table 1. 

**Table 1:** Clinical status and outcomes of the three cases. GA: gestation age; hsPDA: hemodynamic significant patent ductus arteriosus; NIV: non-invasive ventilation; HFOV: high-frequency oscillatory ventilation; VG: volume guarantee; PDA: patent ductus arteriosus; LA/Ao: left atrium to aorta; *Incomplete Paracetamol: premature discontinuation of the paracetamol regimen due to acute kidney injury.

Feature	Case 1	Case 2	Case 3
GA / Birth Weight	24 weeks / 740g	24 weeks / 835g	27 weeks / 1000g
Indication	hsPDA, NIV dependent	hsPDA, medical failure	hsPDA, medical failure
Ventilation prior procedure	HFOV + VG	HFOV + VG	HFOV
Medications	2 courses Paracetamol	Incomplete Paracetamol*	2 courses Paracetamol
PDA size	3.0mm	2.3mm	3.0mm
PDA length	7.5mm	9.6mm	6.0mm
LA/Ao Ratio	1.6	1.8	1.7
Device Used	Piccolo 5/2	Piccolo 4/2	Piccolo 5/2
Age / Weight at Procedure	Day 36 / 1.0kg	Day 31 / 1.05kg	Day 32 / 1.28kg
Primary Outcome	Extubated Day 8 post-procedure	Extubated Day 15 post-procedure	Persistent HFOV
Final Status	Success / Discharged	Success / Discharged	Deceased (Day 96 - Sepsis)

## Discussion

Hemodynamically significant patent ductus arteriosus (hsPDA) in extremely low birth weight (ELBW) infants remains a complex therapeutic challenge. PDA can result in pulmonary overcirculation and systemic hypoperfusion. It is associated with increased mortality and morbidities such as bronchopulmonary dysplasia, pulmonary hemorrhage, prolonged ventilation, intraventricular hemorrhage, periventricular leukomalacia, and necrotizing enterocolitis [5,6].

Management of patent ductus arteriosus (PDA) in preterm infants has been a challenging and controversial topic for neonatologists and cardiologists. Although medical therapy remains first-line treatment, its efficacy is variable and often suboptimal; closure rates for commonly used agents such as indomethacin, ibuprofen, and acetaminophen range from 45% to 76% after a single course. Consequently, repeated courses are often needed to achieve higher closure rates [7]. Surgical PDA ligation is usually only considered when medical treatments have either failed or are contraindicated [8]. However, accumulating evidence suggests that surgical ligation in ELBW infants is associated with significant morbidity. The most common short-term complication is vocal cord paralysis due to recurrent laryngeal nerve injury [9]. Additionally, surgical ligation is associated with recognized complications, including post-ligation cardiac syndrome, chylothorax, surgical site infection, and concerns regarding potential adverse neurodevelopmental outcomes. Furthermore, transporting preterm infants to the operating room or cardiac catheterization laboratory may expose them to additional physiological instability.

The advent of transcatheter PDA closure using low-profile devices, particularly the Amplatzer Piccolo Occluder, has significantly altered the treatment landscape. The pivotal multicenter trial by Sathanandam et al. demonstrated a procedural success rate of 95.5% in infants ≥700 g, with low rates of major adverse events [10]. Subsequent real-world studies have reported comparable success rates (90-98%) and acceptable safety profiles in infants weighing less than 1.5 kg [11-13].

In line with these reports, our small series demonstrated successful bedside transcatheter PDA occlusion in all three ELBW infants. Each infant achieved immediate, complete ductal occlusion without complication. Two of our three infants demonstrated clinically meaningful respiratory improvement and were successfully extubated within two weeks of PDA closure. This observation is consistent with previous reports suggesting that transcatheter closure may facilitate ventilator weaning by reducing pulmonary overcirculation and improving lung compliance. 

One infant in our series died due to progressive gastrointestinal complications and nosocomial sepsis. Importantly, there was no echocardiographic evidence of device-related obstruction or hemodynamic instability preceding clinical deterioration. Patent ductus arteriosus is an independent risk factor for the development of necrotizing enterocolitis in very low birth weight infants [14]. However, current evidence does not demonstrate a causal relationship between transcatheter PDA closure and increased NEC risk. 

Bedside transcatheter PDA occlusion offers a distinct advantage by overcoming both the physiological and logistical constraints of conventional catheterization. In our setting, cath lab availability is highly limited. When combined with the fact that ELBW infants are susceptible to infection and sudden hemodynamic instability, safely timing transfer to the cath lab becomes difficult. By performing the procedure at the bedside, the intervention is driven entirely by clinical necessity rather than facility scheduling, allowing the infant to remain undisturbed.

This bedside approach also helps address important resource and geographical limitations. Dependence on fixed catheterization laboratory availability may lead to avoidable delays in treatment. Training cardiologists to perform these procedures at the bedside enables a more flexible, cluster-based model of care, reducing the need to transfer fragile neonates from district hospitals to tertiary centers. In addition, echo-guided bedside procedures significantly reduce and eliminate the exposure to ionizing radiation, which is an important safety consideration for ELBW infants.

However, bedside echocardiography-guided PDA closure is not without challenges. The NICU environment is often crowded with incubators, ventilators, and multiple lines, making procedural setup difficult. Procedural success depends heavily on close coordination between the interventional cardiologist and the echocardiographer. Compared with fluoroscopy, echocardiography provides a more limited field of view, requiring continuous image optimization without applying excessive pressure to the infant’s chest, which may precipitate cardiorespiratory instability. The operator must rely entirely on ultrasound guidance to advance wires and deploy the device safely, while minimizing the risk of complications such as vascular injury or device embolization. Consequently, the learning curve for a fully echo-guided technique remains steep.

Despite promising short-term outcomes, important questions remain regarding the optimal timing of intervention, long-term pulmonary outcomes, and neurodevelopmental impact. Current literature largely consists of observational studies and registry data. Randomized trials comparing transcatheter closure, surgical ligation, and conservative management in preterm infants are still needed. 

## Conclusions

Our small case series adds to the evidence suggesting that bedside echocardiography-guided transcatheter PDA closure is a feasible alternative to surgical ligation in selected ELBW infants. Performing the procedure at the bedside may reduce the risks associated with neonatal transport and could support respiratory recovery in this particularly vulnerable group.

In Malaysia, where pediatric cardiothoracic surgical services are limited in specific tertiary centers, bedside PDA device occlusion represents an evolving alternative that may bypass the complexities of inter-hospital transfer. While our small case series limits broad conclusions, these findings suggest that structured bedside programs could potentially mitigate the inherent risks for unstable neonates. As this technique gains local traction, systematic data collection via multicenter registries will be vital to better define procedural safety and long-term outcomes, with the goal of more equitable access to PDA management nationwide.
